# Assessing energy and environmental impacts of Turkish-style tea brewing: efficiency, consumption, and carbon footprint

**DOI:** 10.1038/s41538-025-00405-y

**Published:** 2025-05-09

**Authors:** Oktay Zorlu, Bedirhan Güneşdoğmuş, Sefa Sözer, Selman Akaslan, Batuhan Göçen, İlker Dikmen, Ümit Ünver

**Affiliations:** 1 Yalova University, Engineering Faculty, Yalova, Turkey; 2 Korkmaz Kitchenware R&D Center Tepeören, İstanbul, Turkey; 3 Barbaros Mah. Mor Sümbül Sk, Ataşehir/İstanbul, Turkey

**Keywords:** Science, technology and society, Environmental impact

## Abstract

Energy efficiency in food preparation is a critical yet often overlooked aspect of sustainability. Despite tea being one of the most consumed beverages worldwide, research on the energy efficiency of its brewing process—particularly at the household level—remains limited. This study addresses this gap by investigating the energy cost, efficiency, and environmental footprint of Turkish-style tea brewing, a method characterized by its unique double teapot system and prolonged steeping process. Experimental tests were conducted on a standard kitchen stove using three burner sizes and varying flame modes. Energy efficiency, analyzed using the First Law of Thermodynamics, ranged from 31% to 70%, with specific energy consumption between 0.53 and 0.93 kJ/kg. Results reveal a trade-off between energy efficiency and brewing time, highlighting the need for optimized techniques to reduce energy waste. Given the massive global tea consumption, this study provides valuable insights for future research on sustainable and energy-efficient food preparation practices.

## Introduction

Tea is one of the most consumed beverages across many cultures and in daily life^1^. Due to high per capita tea consumption in Türkiye, numerous scientific studies have focused on the various sides of tea^2^. As a detailed continuation of previous studies^3^, this study includes an energy analysis of tea brewing.

Tea is valued for both its taste and health benefits. Rich in antioxidants, tea can help combat free radicals in the body, reducing cellular damage and potentially lowering the risk of various chronic diseases^4^. The catechins in green tea contribute to weight management by boosting metabolism^5^. Rich in antioxidants and catechins, tea offers numerous health benefits, including, improved heart health, enhanced cognitive function, and boosted immunity^6,7^. Additionally, green tea may help manage stress through its calming effects^8^. When consumed in moderation, tea can be a pleasant beverage that contributes to health^9^. However, as with many things, excessive tea consumption should be avoided^10^.

Numerous studies have investigated various aspects of tea. such as the total phenolic content of dried and brewed tea^11^, the transfer of nitrate during brewing^12^, quality characteristics at different brewing times^13^, potential anti-cancer effects^14^, and antioxidant capacities^15^.

There are various types of tea around the world^16^ and they can be categorized into three main varieties based on morphology: Chinese tea, Assam tea, and Cambodian tea. Numerous hybrids have emerged from these varieties^17^ of which black tea and green tea are the most commonly consumed types worldwide.

In Türkiye, black tea, the most frequently consumed type, contains caffeine and minerals such as sodium, potassium, calcium, and magnesium, contributing to its stimulating effects^18^. From a health perspective, green tea is known to have a protective effect against neurodegenerative diseases like Alzheimer’s and Parkinson’s^19^.

Some studies indicate that tea is not merely a beverage; it is also a medium through which social connections are forged, sharing occurs, and cultural rituals are experienced^20^. In various cultures around the world, drinking tea is not only a habit but also symbolizes communication and solidarity within communities^21^. While tea was a pleasant pastime in its homeland, China, it has evolved into a cultural phenomenon in Japan as well^22^.

Tea is consumed everywhere in Türkiye in daily life, from homes to workplaces, from school canteens to offices. It is observed that manufacturers in Türkiye are generally small family-owned businesses^23^. According to the World Tea Report 2016^24^ data, tea production areas in the world reached 4.52 million hectares^25^. Tea production increased by 4.2 percent compared to 2015 and reached 5.31 million tons. While China ranks first in world tea production with 2 million 270 thousand tons, Türkiye ranks fifth with 260 thousand tons^26^. Türkiye ranks first in the world in annual tea consumption per capita with 3.5 kilograms^27^. As such the Turkish tea market naturally attracts the attention for investors^28^. In Türkiye, nearly 245 million glasses of tea is consumed every day that makes the energy consumed for tea more considerable^3,27^. Considering that approximately 3 grams of dry tea are used per cup of brewed tea, Türkiye’s daily dry tea consumption is estimated to be around 373 tons. This value aligns with the country’s annual per capita tea consumption statistics. Despite such high tea consumption in the country, there are no published studies on the energy cost of tea brewing.

Tea has become a key subject in sustainability and energy efficiency studies due to its widespread global consumption^29^. In the literature, various energy analyses have been conducted on different aspects of tea production, including the integration of renewable energy into tea processing^30^ and exergy performance assessments^31^. However, all these studies have focused on the production stages of tea, up until it reaches the final consumption point. None of them have specifically examined the energy analysis of tea brewing, which represents the consumption stage.

In this study, Turkish-style tea brewing, which differs from other tea traditions and preparation methods, is examined. Although similar brewing experiments exist in the literature^32^, most studies have focused on the energy costs^33^, environmental impacts^34^, and energy efficiency^35^ of kitchen stoves. These studies have generally been conducted following the Water Heating Test (WHT) standard^36–38^. However, none of them have specifically applied Turkish-style teapots or brewing procedures. This study addresses this gap by providing an energy analysis of Turkish-style tea brewing.

This study is the first to examine the energy efficiency, specific energy consumption, and energy cost of Turkish type tea brewing. It also provides valuable data for estimating the total energy use and carbon footprint of tea brewing in Türkiye from an engineering perspective. The significance of this research lies in its focus on the energy cost and efficiency of Turkish-style tea brewing, in a country with one of the highest per capita tea consumption rates in the world. By presenting experimental results, this study fills an important gap in the fields of environmental sustainability and energy efficiency in food production.

## Results

In this study, the energy efficiency of the Turkish-style tea brewing process was investigated experimentally. The key parameters of the experiments affecting energy efficiency were determined by ANOVA analysis. The results were explained and discussed using the notation provided below. The burner sizes used in the experiments were classified as large (LB), medium (MB), and small (SB). For each burner, six different experiments were conducted for both low flame (LF) and high flame (HF) modes. To increase the reliability of the results, each experiment was repeated eight times. Excluding the highest and lowest values, the remaining six measurements were used for the analysis. The energy efficiencies of all experimental modes were calculated, and the notation used is presented in Table 1.

**Table 1:** Experiment types and dimensions

	Small burner SB	Medium burner MB	Large burner LB
Burner diameter	55 mm	70 mm	100 mm
Low flame diameter LF	60 mm (SBLF)	80 mm (MBLF)	110 mm (LBLF)
High flame diameter HF	80 mm (SBHF)	105 mm (MBHF)	140 mm (LBHF)

**Fig. 1:**
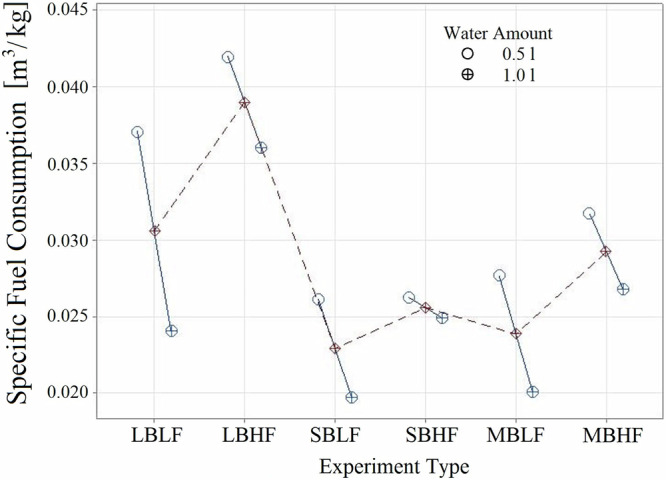
Effects of experiment type and water amount on specific fuel consumption.

The results of the analysis of variance (ANOVA) for natural gas consumption in the tea brewing process, differentiated by experiment type and water amount, are displayed in Fig. 1. It was observed that, in all experiment types, the specific fuel consumption for boiling 1 L of water was lower than that required for boiling 0.5 L (Fig. 1). Experiments conducted with the large burner (denoted by experiments starting with “LB”) consume more energy compared to those conducted on other burners. Additionally, in all experiments conducted more energy was consumed in the high flame mode compared to the low flame mode with the same burner.

In experiments conducted at high flame mode, the burner power (which corresponds to the rate of energy consumption) was higher than the rate of heat transfer to the water in the teapot. Furthermore, in the case of the large burner, when using high flame mode, the flame extending beyond the edges of the teapot significantly reduces energy efficiency. Due to the extension of the flame beyond the teapot’s base, a substantial amount of energy is dissipated into the ambient air, reducing the energy available for water heating. In other burners, higher energy consumption was observed in high-flame mode experiments due to greater losses and leaks.

Table 2 shows the amount of natural gas consumed per unit of water (m^3^/kg), along with the average value (FIT), standard error (SE), and 95% confidence interval (95% CI) for the selected parameters. For the SBLF experiment type, which consumes the least amount of fuel for tea brewing, the confidence interval indicates that with 95% probability, the fuel consumption could range between 0.0173241 and 0.0219037 m^3^/L. In contrast, for the LBHF experiment type, which showed the highest fuel consumption, the natural gas consumption is estimated to range between 0.0334158 and 0.0379953 m^3^/L with 95% probability.

**Table 2:** Model prediction

Experiment no.	Experiment type	Fit	SE Fit	95% CI	95% PI
1	SBHF	0.0222806	0.0011465	(0.0199908; 0.0245703)	(0.0145882; 0.0299729)
2	SBLF	0.0196139	0.0011465	(0.0173241; 0.0219037)	(0.0119216; 0.0273062)
3	LBLF	0.0272722	0.0011465	(0.0249825; 0.0295620)	(0.0195799; 0.0349645)
4	LBHF	0.0357056	0.0011465	(0.0334158; 0.0379953)	(0.0280132; 0.0433979)
5	MBLF	0.0205556	0.0011465	(0.0182658; 0.0228453)	(0.0128632; 0.0282479)
6	MBHF	0.0259389	0.0011465	(0.0236491; 0.0282287)	(0.0182466; 0.0336312)

**Fig. 2:**
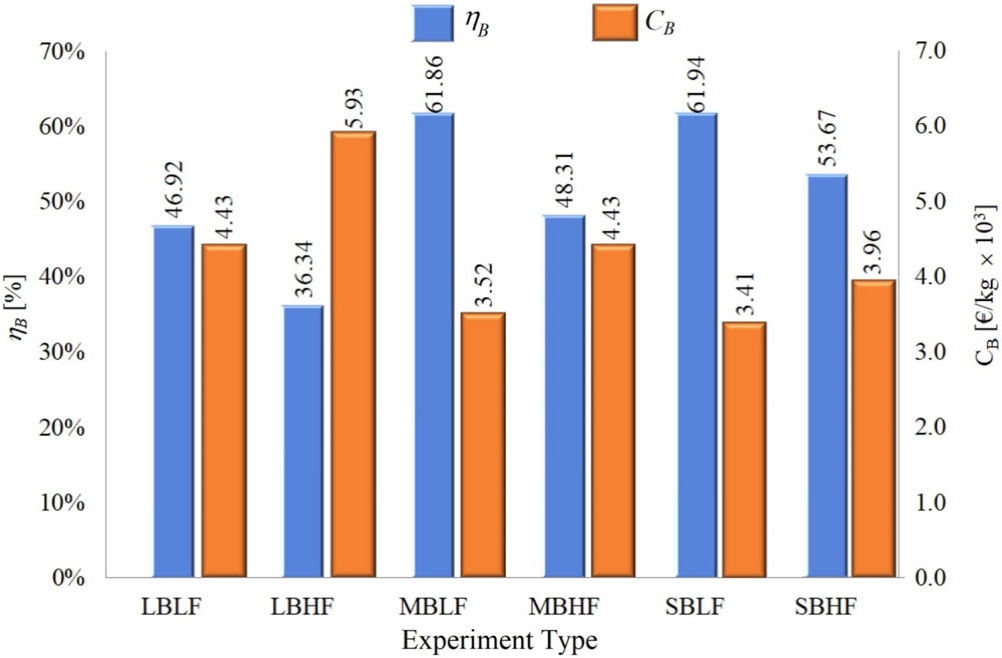
Tea brewing efficiency and tea brewing cost.

Figure 2 presents the mean efficiency (ηB) and monetary cost (CB) of tea brewing. The most efficient boiling process (61.94%) occurred on the small burner (SB), resulting in a cost of 3.41 €/kg × 10^−3^, which is slightly lower than that of the middle burner (MB). Both burners operated in low flame (LF) mode. As expected, the highest cost of Turkish-style tea brewing was observed under the lowest efficiency conditions (36.34%), calculated as 5.93 × 10^−3^ €/kg. It was observed that flame diameter and efficiency are inversely proportional, which aligns with the ANOVA analysis.

**Fig. 3:**
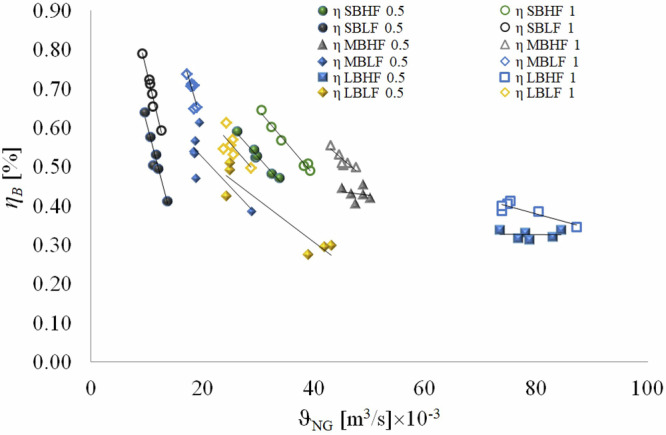
Efficiency versus natural gas consumption.

Figure 3 presents the efficiency–natural gas consumption relationships for all experiment types. Each experiment type is represented with different symbols. The results of the six experiments conducted for each experiment type are presented. In each experiment type, either the fully open (highest) or the lowest setting of the kitchen stove was used to keep the natural gas flow constant. However, even the finest settings of the kitchen stove resulted in significantly varying natural gas flow in each experiment. When analyzing the energy efficiency of cooking, even if the setup is exactly the same, the gas flow changes every time. Therefore, for the experiments to determine the cooking energy efficiency, both gas consumption and processing time must be measured precisely. However, Fig. 3 clearly demonstrates that variations in natural gas flow significantly impact energy efficiency. Furthermore, regardless of the experiment type, energy efficiency consistently increases with the amount of tea brewed.

**Fig. 4:**
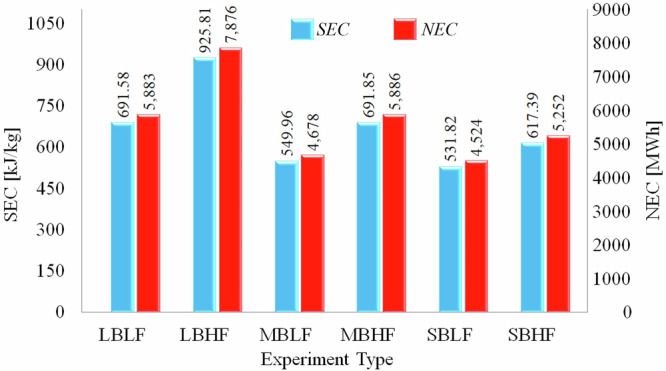
Specific energy consumption and carbon footprint of tea brewing.

The specific energy consumption (SEC) and national energy consumption (NEC) results for tea brewing are shown in Fig. 4. Parallel to the efficiency results, the lowest specific energy consumption was achieved 532 kJ/kg on the small burner at low flame mode. The SEC values obtained in this study are in agreement with Hager (2013). It must be noted that, the SEC given in between 551 kJ/kg and 1145 kJ/kg are given for heating 0.5 L and 1 L of water using electric heaters^39^. However, the findings of this study are slightly higher than^33^. The SEC values given by Anozie et al. are for heating 2.25 L of water. This supports the conclusion we stated at the beginning of the section: “less energy per unit mass of water is required to heat more water compared to heating less water.”

**Fig. 5:**
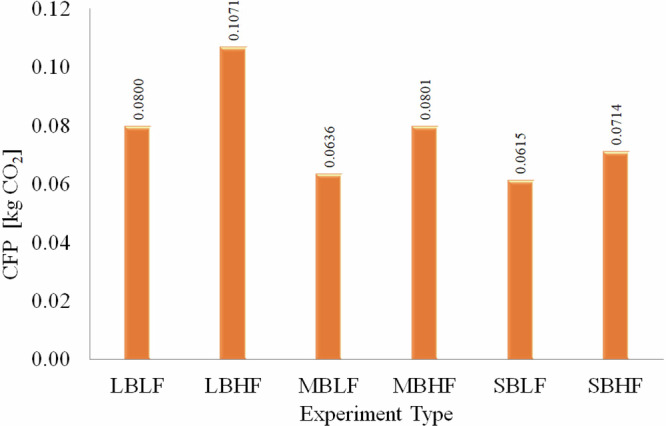
Carbon footprint of tea brewing.

Figure 5 illustrates the CO₂ emissions resulting from natural gas consumption, providing readers with an understanding of the environmental impact of tea brewing. The values presented in the figure represent the CO₂ emissions associated with brewing 1.5 kg of tea. The lowest carbon footprint, recorded at 0.0615 kgCO₂, corresponds to the SBLF brewing conditions. Similarly, as observed in the SEC and NEC graphs, the highest emission value of 0.11 kgCO₂ was found in the LBHF experiment type. Considering that a single glass of Turkish tea weighs approximately 0.125 kg and an estimated 245 million glasses of daily national consumption is reported, Turkey’s daily tea consumption contributes to an estimated 1255 tons of CO2 emissions. This highlights the significant, yet often overlooked, environmental impact of daily rituals. Based on these findings, promoting a more sustainable tea brewing process requires raising public awareness about reducing carbon emissions. In particular, consumers should be encouraged to avoid brewing tea with large-sized burners at high flame mode (LBHF), as this significantly increases CO₂ emissions.

## Discussion

The results of this study reveal that burner size, flame mode, and the amount of tea brewed are important parameters for a given teapot size in Turkish-style tea brewing. For more energy-efficient brewing, large burners and high flame modes should be avoided. The analysis results suggest that the most efficient tea brewing method is to use a small or medium burner at low flame modes. Furthermore, the energy consumption range observed in the LBLF experiment is wider than in all other experiments. The large energy consumption range between the 0.5 L and 1 L tea brewing experiments in the LBLF experiment could be due to higher losses per unit mass.

In Türkiye, tea is brewed daily in almost every household and workplace. Based on the lowest and highest energy costs of tea brewing, the total energy cost for the country’s tea consumption is estimated to range between approximately 125 k€ and 218 k€ annually. In other words, increasing tea brewing efficiency from 36.34% to 61.94% —a 25.6% improvement—leads to a cost reduction of about 42.5%. This means that optimizing tea brewing efficiency could reduce Türkiye’s daily energy expenditure on tea brewing by approximately 93 k€ annually. Implementing an appropriate energy efficiency strategy, informing the public, and raising awareness could contribute to significant cost savings.

It is important to note that there is strong agreement between our results and those reported in the existing literature, as presented in Table 3 when compared with the outcomes of Fig. 3. When the results from the existing literature are plotted, they fall within the overall range of our findings. This close alignment provides compelling evidence that the results of this study offer a comprehensive representation of existing knowledge in this area, effectively encompassing and extending the findings previously reported in the literature.

**Table 3:** The results from similar literature

Literature	Condition	Efficiency (%)
Ko and Lin^42^	Experimental	32.0–38.0
Anozie et al.^33^	Experiments repeated 3 times. 2.25 l’s of water boiled.	73.0
Ayub et al.^45^	Review	45.0–60.0
Joshi and Waghole^46^	Experiments (variable utensil dimensions)	48.2–61.5
Deymi-Dashtebayaz et al.^34^	Experimental	64.0–65.6

Assuming that a glass of tea weighs 0.125 kg and that Türkiye consumes an average of 245 million glasses of tea daily, the national energy consumption for tea brewing ranges between 4524 kWh and 7876 kWh each day. This is equivalent to the energy output of a power plant with a capacity of 189 MW under low consumption and 328 MW under high consumption. The 3252 MWh difference in energy usage between these two scenarios highlights the critical importance of energy efficiency in tea brewing. This significant variation demonstrates the need for a comprehensive study of the ‘most efficient tea brewing method’ at a national level in Türkiye. By considering factors like stove types, cooking appliances, and teapot designs, a nationwide ‘Best Practices’ guide for tea brewing could be developed, maximizing energy efficiency in tea consumption.

Finally, this study demonstrates that promoting energy-efficient tea brewing methods in Türkiye can lead to significant energy savings through sustainable food processing. Future research is recommended to explore the detailed environmental impacts of tea brewing, including the use of induction stoves, resistance-based electric stoves, and electric tea machines, as well as teapot geometries and the thermal performance of tea brewing equipment. This study has determined that medium and small burners should be preferred in the lowest possible flame mode for tea brewing. By raising public awareness of energy-efficient tea brewing, a significant portion of the 3252 MWh daily energy consumption difference between efficient and inefficient brewing methods can be conserved nationwide.

## Methods

In this study, the factors affecting the energy consumed during tea brewing was investigated experimentally. Quality-related outcomes, such as tea color, taste, and concentration, were not considered in the analysis.

The experimental procedure was applied in a manner similar to WBT Version 4.2.3^40^. However, WBT is a standard designed for the thermal and emission analysis of kitchen stoves. In this study, parameters such as stove efficiency and emission distribution were excluded from the scope. Instead, assuming complete combustion of natural gas, CO₂ emissions resulting from natural gas consumption were calculated. To maintain the study’s focus on the energy analysis of Turkish-style tea brewing, the analyses were conducted using the First Law of Thermodynamics by measuring the energy required to boil water, the elapsed time, and natural gas consumption.

At the beginning of each experiment, a constant volume of 0.5 liters of water was used for the initial boiling and brewing processes, and then 1 liter water was used for the second heating. The time elapsed and natural gas consumption were measured throughout the process.

Within the scope of the research, common household products such as household cookers, teapots and tap water were used in the experiments. Temperatures were measured with a ± 0.5% C PCE-T390 thermologger, and time was measured with a chronometer.

### Experiment procedure

**Fig. 6:**
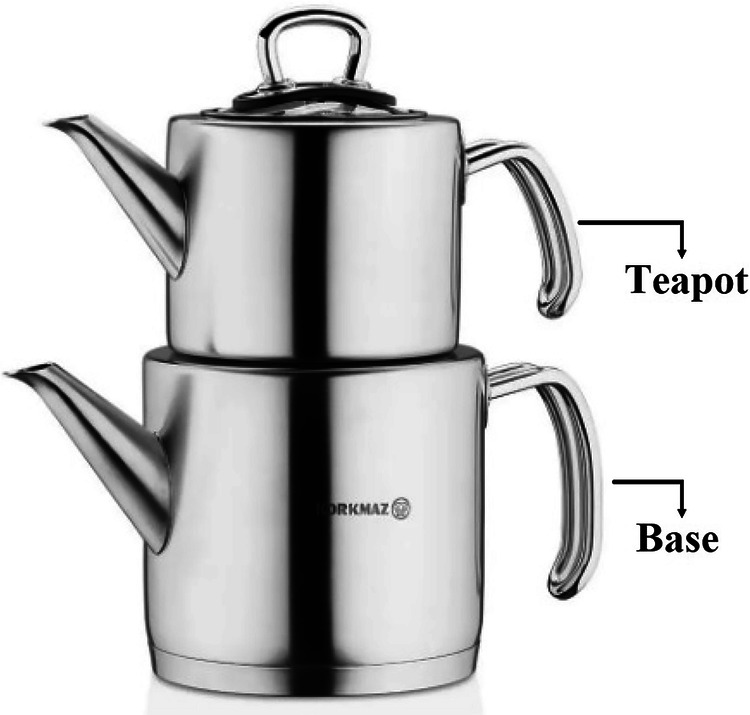
Traditional Turkish type teapot.

The calorific value of the natural gas used as the energy source in the experiment was calculated based on the data specified by the energy distribution company in accordance with ISO 6976 and ASTM D3588 standards. The experiment was conducted on a natural gas stove tested in accordance with the EN 437 standard. A typical traditional Turkish tea kettle, as shown in Fig. 6, consists of two parts. The upper part is used only for brewed tea and is called the teapot. The lower part, which is used only for hot water, is referred to as the base in this study. The volume of the base is twice that of the teapot. This type of tea brewing typically takes at least 20 minutes. During this process, the lower compartment, which contains boiling water, helps maintain a high temperature, as Turkish tea is traditionally served hot. The dual-compartment design of the teapot efficiently preserves and regulates heat, ensuring that the temperature remains consistently high throughout the brewing and serving process.

In the experiments, 0.5 L of water is first placed in the base. Once the water reaches the boiling temperature, it then is transferred to the teapot. Dry tea leaves (referred to as tea) are then added when the water temperature drops to 80 °C, and the mixture is allowed to brew over the base. Meanwhile, 1 L of water is added to the base and heated to the boiling temperature again. This process is referred to as tea brewing. Normally, when the base is filled with water in the second step, it is already hot. However, in the experiments, the base was cooled to the ambient temperature by adding water, in order to maintain consistency. The natural gas meter and temperature were measured and recorded immediately before and after each boiling cycle. The duration of the process was tracked using a timer.

The scope of this study is to determine the energy consumption and CO₂ emissions of Turkish-style tea brewing. Special attention was given to maintaining the focus on energy efficiency. The efficiency and waste of kitchen stoves were excluded from the study’s scope. Therefore, a slightly modified procedure, different from the Water Boiling Test (WBT), was applied to align with the Turkish-style tea brewing process. The experiment focused on measuring the duration, the energy used to heat the water, and the consumption of natural gas. Additionally, the following assumptions were made:

i. The study’s scope is limited to the efficiency of the Turkish-style tea brewing process. Heat losses due to natural convection and radiation from hot surfaces were excluded.
ii. In the experiment, 0.5 L of water was first boiled to brew tea, followed by boiling 1 L of water to complete the test. Before boiling the 1 L of water, the kettle base was cooled to room temperature.
iii. The experiments continued until the water in the kettle reached 97.8 °C.
iv. Energy losses due to water evaporation were neglected.
v. Natural gas pollutants were considered negligible, and complete combustion was assumed^41^. Thus, only CO2 was considered as a combustion product, while other greenhouse gases like N2O and CH4 were excluded.
vi. Since the focus of the study is the energetic analysis of brewing tea, fuel composition, combustion temperature, and other combustion conditions are not taken into consideration.
vii. At the end of the experiment, it was assumed that all liquid inside the kettle was at the same temperature.
viii. The elapsed time during the experiment was measured manually, with an accepted margin of error of ±1 second.

### Governing equations

In this study, each type of experiment was repeated at least eight times. The highest and lowest values were excluded, and the averages were calculated for each experiment type. This means that each result is based on six experiments. The energy transferred to water was given by Lombardi (2016)^37^ similarly to Eq. (1). The equation is similar to Aier’s (2022) equation^36^. But in this study, the boiled water is not taken into consideration as given in ref. ^42^.

1 $${Q}_{w}={m}_{w}.{C}_{p}.({T}_{w,b}-{T}_{w,s})$$

where *Q*_*w*_ is the thermal energy transferred to the water until boiling starts (kJ), *m*_*w*_ is the mass of water used for tea brewing, which is 1.5 kg in this case (kg), *C*_*p*_ is the specific heat of water (kJ/kg °C), ${T}_{s,w}$ is and ${T}_{b,w}$ are the initial temperature of the water (measured at the start of the experiment, °C), and the boiling temperature of the water (°C) respectively.

The energy consumed by burning natural gas for this process was given similar to Eq. (2)^32^.

2 $${Q}_{{ng}}={\mathcal{V}}.\rho .{LHV}$$

where ${Q}_{{ng}}$ represents the thermal energy consumed by burning natural gas (kJ), ${\mathcal{V}}$ is the volume of the natural gas measured by the natural gas meter (m^3^), $\rho$ and LHV are the density (kg/m^3^), and the lower heating value (kJ/kg) of natural gas provided by the natural gas supplier company.

The thermal power of the process (*P*_*th*_) is given by ref. ^36^ similar to Eq. (3). The equations for calculating the efficiency of the tea brewing process is introduced in several studies as in Eq. (4)^35,37,43^, and it was adopted from^42^

3 $${P}_{th}={Q}_{ng}\,/\,\Delta (t)$$

4 $$\eta =\frac{{Q}_{w}}{{Q}_{{ng}}}$$

here Δ*t* the time elapsed to boil water (s). The consumption rate ($\dot{{\mathcal{V}}}$) was calculated. Detailed information about the analytical procedure employed within this paper can be found in Lombardi^43^. The energy cost of tea brewing was determined using Eq. (6);

5 $$\dot{{\mathcal{V}}}=\frac{{\mathcal{V}}}{{dt}}$$

6 $${C}_{B}=\frac{{\bar{C}}_{{ng}}{\mathscr{.}}{\mathcal{V}}}{{m}_{t}}$$

Where,${\mathcal{V}}$ and $\dot{{\mathcal{V}}}$ represent the consumed natural gas in the experiments (m^3^) and the volumetric natural gas consumption rate (m^3^/s) respectively. ${C}_{{ng}}$ is the price of the natural gas (€/m^3^), *m*_*t*_ is the amount of brewed tea in (kg) and ${C}_{B}$ is the brewing cost (€/kg). Equation (7) was presented by ref. ^43^ to calculate the Specific Energy Consumption (SEC, kj/kg). NEC, represents the national energy consumption for tea brewing (kWh), and is calculated using Eq. (8). In this equation, *m*_*g*_ represents the mass of a single glass of tea, TC represents the total national tea consumption (per glass), as reported by the Trabzon Commodity Exchange, and k is the unit conversion factor from kJ to kWh.

7 $${SEC}=\frac{{Q}_{{ng}}}{{m}_{w}}$$

8 $${NEC}=\frac{{Q}_{{ng}}}{{m}_{g}}\cdot \sum {TC}\cdot k$$

While calculating the carbon footprint, (Eq. 8) follows the procedure outlined in WBT Version 4.2.3. However, WBT is a standard designed for the thermal and emission analysis of kitchen stoves. Nevertheless, the focus of our study is not the analysis of kitchen stoves but rather the analysis of the tea brewing process. Therefore, only the carbon emissions resulting from the natural gas consumption used specifically for the brewing process were calculated. In Eq. (9), *Fp* is the coefficient representing the pollutant factor (kg/m^3^) from the CO_2_ produced by natural gas combustion.

9 $${CFP}={F}_{p}\cdot {\mathcal{V}}$$

The uncertainty analysis was conducted for efficiency, specific fuel consumption, and CO_2_ parameters using Eq. (10). In the equation, ${x}_{j}$ represents the error rate of jth independent variable, while δY/δx denotes the derivative of the parameter equation with respect to the independent variable.

10 $${dY}=\sqrt{\left\{\mathop{\sum }\limits_{j=1}^{n}{\left(\frac{\delta Y}{{\delta x}_{j}}{x}_{j}\right)}^{2}\right\}}$$

The accuracies of the devices used in the study and the calculated uncertainties are presented in Table 4 and Table 5, respectively. Uncertainty is calculated by using Eq. (10) given by refs. ^29,44^;

**Table 4:** Accuracies of measurement devices

	Gas flowmeter	Thermal data logger	Timer	Mass
Measuring accuracy	±0.2%	±2.2 °C	±1 s	±25 g

**Table 5:** Relative uncertainty

	*η*	SEC	CO_2_
Relative uncertainty	4.70%	0.25%	0.25%

### ANNOVA analysis

In the experiments, both the size of the burner and the natural gas valve setting were defined as experimental parameters. Among the parameters studied, it was hypothesized that indoor air temperature (T_i_), outdoor air temperature (T_o_), temperature difference (ΔT = T_b,w_ – T_s,w_), water amount (*m*_*w*_), and the type of experiment could all influence the tea brewing efficiency (*η*_*B*_). The analysis started by investigating which of these five factors had the greatest effect on energy consumption. According to the results of the first analysis, as shown in Table 6, the p-values for the T_o_ and T_i_ parameters were found to be greater than 0.05. A p-value greater than 0.05 indicates that these parameters fall within the 5% region at both the beginning and end of the normal distribution curve. This suggests that these parameters have no statistically significant effect on the result, or their effect is negligible. Therefore, parameters with p-values greater than 0.05, which can be considered as confounding variables, were removed from the model, and the analysis was repeated.

**Table 6:** Analysis of variance

Analysis no.	Source	DF	Adj SS	Adj MS	*F* value	*P* value
I.	Regression	9	0.002970	0.000330	25.56	0.000
	ΔT	1	0.000062	0.000062	4.83	0.032
	T_o_	1	0.000008	0.000008	0.63	0.429
	T_i_	1	0.000019	0.000019	1.48	0.229
	m	1	0.000761	0.000761	58.95	0.000
	Experiment type	5	0.002012	0.000402	31.16	0.000
II.	Experiment type	5	0.000625	0.000125	11.39	0.001
	m	1	0.000093	0.000093	8.45	0.016
	ΔT	55	0.000769	0.000014	1.28	0.357
III.	Experiment type	5	0.002118	0.000424	31.33	0.000
	m	1	0.000774	0.000774	57.21	0.000

In the first analysis, the model summarized in Table 7 shows an adjusted R^2^ value of 75.69%. This indicates that the study provides useful insights and supports the potential for establishing a relationship between the selected parameters and tea brewing efficiency. However, the relatively low R^2^ value suggests that the model’s ability to represent the data is only marginally acceptable.

**Table 7:** Model summary

Number of analysis	S	*R*^2^	*R*^2^(adj)
I	0.0035934	78.77%	75.69%
II	0.0033118	97.09%	79.35%
III	0.0036771	76.69%	74.54%

When the T_o_ and T_i_ parameters were removed and the analysis was repeated, it was found that the p-value for the ΔT parameter was also greater than 0.05, indicating that it did not affect the result. While ΔT appeared to be a significant parameter in the first analysis, it was deemed ineffective in the second analysis and was considered a confounding variable. In Table 7, the R^2^ value for the second analysis was calculated as 79.35%, which is higher than the previous analysis and provides insight into the magnitude of the effect of confounding variables on tea brewing efficiency.

In the third analysis, where the ΔT parameter was also removed, it was found that the p-values for the experimental type and water amount parameters were both 0, indicating that these two parameters directly affect tea brewing energy efficiency. The R^2^ value for the third analysis, shown in Table 7, is 95%, suggesting with 74.54% confidence that these two parameters are highly representative of the results.

## Data Availability

The data supporting the findings of this study are available and can be shared upon request.
